# Factors influencing coronary artery target lesion revascularization after drug-coated balloon angioplasty

**DOI:** 10.3389/fcvm.2024.1387074

**Published:** 2024-05-16

**Authors:** Alberta Claudia Undarsa, Aninka Saboe, Badai Bhatara Tiksnadi, Mohammad Rizki Akbar, Achmad Fauzi Yahya

**Affiliations:** Department of Cardiology and Vascular Medicine, Faculty of Medicine, Universitas Padjadjaran—Dr. Hasan Sadikin General Hospital, Bandung, Indonesia

**Keywords:** drug-coated balloon (DCB), intravascular imaging, restenosis, target lesion revascularization, percutaneous coronary intervention

## Abstract

**Background:**

Concerns regarding restenosis after treatment with drug-coated balloons (DCB) remain. We aimed to identify the incidence of target lesion revascularization (TLR) and explore clinical, procedural, and other factors influencing it.

**Methods:**

Single-center retrospective analysis of a prospective cohort PCI registry study included 80 patients (100 lesions) who underwent successful DCB angioplasty between January 2020 and October 2023 and follow-up angiography within 2 years of either planned or unplanned reason. Incidence and factors associated with TLR were analyzed.

**Results:**

Angiographic evaluation was conducted within a median of 151 days (interquartile range: 109 days). During index procedure, 54% were complex lesions. Intravascular imaging (IVI) was performed in 80% of lesions. TLR occurred in 11% of the lesions and was less frequent in the IVI group compared to the angiography-alone group [6.3 vs. 54.5%; odds ratio: 0.156, 95% confidence interval (CI): 0.042–0.580; *p* = 0.002]. No association was found between baseline and lesion characteristics, lesion complexity, plaque morphology, pre-dilatation procedure balloon type, maximal inflation pressure, or length of DCB between the groups (*p* > 0.05). Multivariate analysis revealed that IVI utilization was independently associated with a lower TLR rate (adjusted odds ratio: 0.116, 95% CI: 0.020–0.669; *p* = 0.016).

**Conclusion:**

In DCB angioplasty, only IVI use exhibited a significant difference in the TLR rate among baseline lesion characteristics and lesion preparation and was independently associated with a lower TLR rate.

## Introduction

Despite the well-established safety and efficacy of drug-eluting stents (DES), the occurrence of in-stent restenosis (ISR) remains a problem, affecting approximately 5%–10% of all percutaneous coronary interventions (PCIs) (1–3). Certain particular settings are linked to increased rates of restenosis and thrombosis following stenting, such as ISR, small-vessel disease, and bifurcation disease, in which stent placement for treatment may not result in effective long-term and mid-term outcomes on follow-up which remains challenging (2, 4). As a result, using drug-coated balloons (DCB) as a treatment approach for coronary arteries to alleviate the stent burden is an appealing option, as it minimizes the need for permanent stent implants, especially in high-risk lesions (3).

The DCB comprises a semi-compliant balloon responsible for the acute mechanical effect in restoring vessel patency during angioplasty. It is coated with an antiproliferative drug that effectively inhibits smooth muscle cell proliferation and migration and is transferred to the vessel wall during balloon inflation (3, 5). The efficacy and safety of DCB in treating in-stent restenosis (ISR) were classified as class IA in the 2018 ESC/EACTS guidelines, similar to native small-vessel disease in randomized controlled trials (RCTs), and other emerging indications, such as high bleeding risk, large-vessel disease, and complex lesions, have also been studied (3, 5).

Despite its promising potential as an alternative to DES, concerns persist regarding restenosis after DCB implantation, and the incidence and potential influencing factors of target lesion revascularization (TLR) still need to be thoroughly assessed (3). Various clinical scenarios have been explored for DCB angioplasty, yielding promising results. However, the specific lesion characteristics impeding favorable angiography outcomes in DCB angioplasty have yet to be established. Several studies have aimed to predict angiographic outcomes after DCB use in specific lesions (e.g., small vessel coronary disease and ISR), but more studies are needed in actual clinical settings (6–8). We hypothesized that identifying clinical characteristics and procedural techniques as predictors of TLR on follow-up angiography could enhance the optimization of revascularization preparation during DCB utilization. Therefore, we conducted a single-center retrospective analysis of a prospective cohort study to investigate the incidence of TLR and evaluate potential influencing factors associated with it.

## Methods

### Study design and population

**Figure 1:**
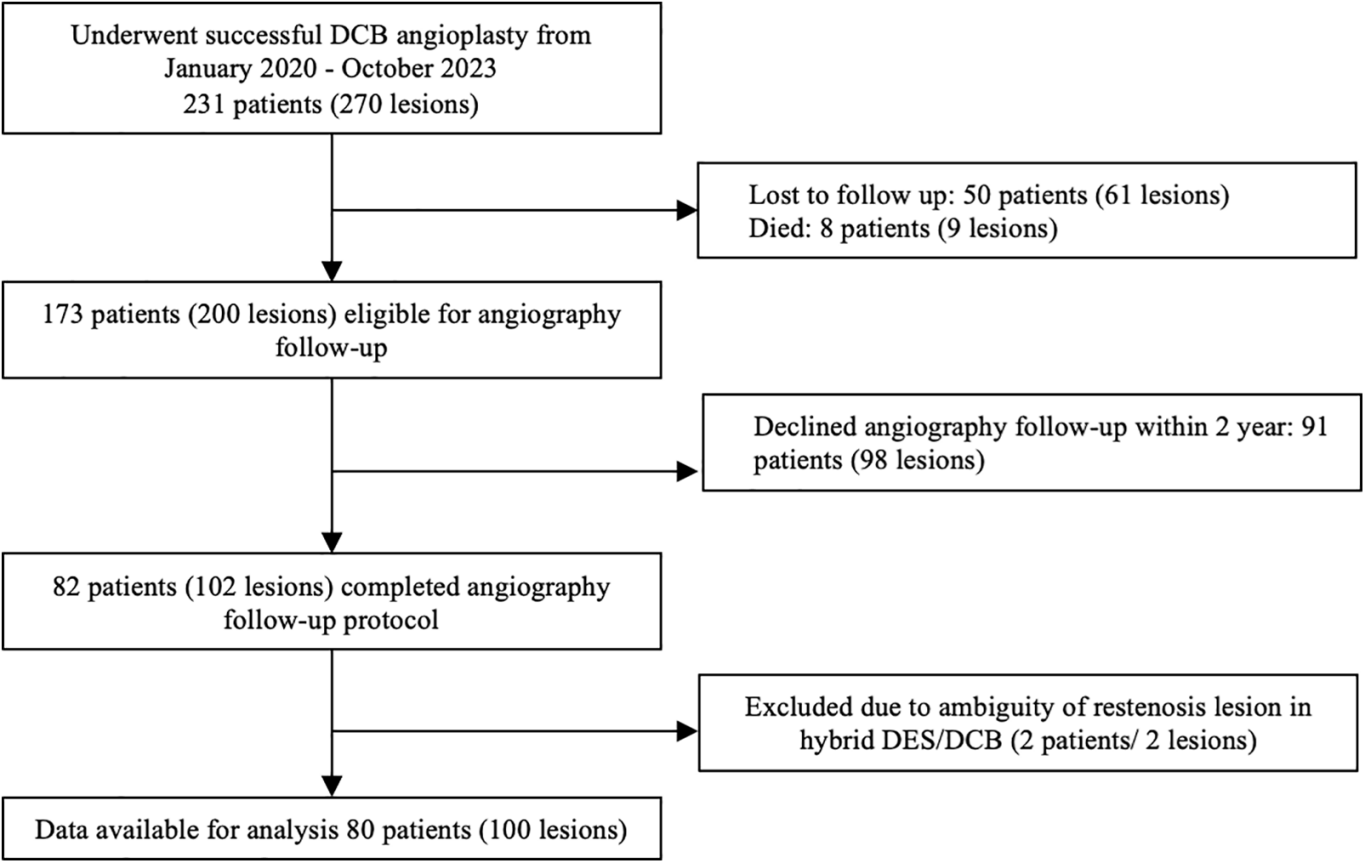
Study flow chart.

This was a single-center retrospective analysis of a prospective cohort PCI registry of Dr. Hasan Sadikin General Hospital involving patients who underwent successful DCB angioplasty. Each patient was enrolled in the DCB PCI registry of Dr. Hasan Sadikin General Hospital, Bandung. Between January 2020 and October 2023, 231 patients with 270 lesions underwent successful DCB angioplasty. As part of the study protocol, all patients were offered follow-up angiography within three months to two years as a planned procedure if no staging procedure was required. Among them, follow-up angiography was performed in 80 patients (100 lesions) as part of the scheduled angiographic evaluation and staged PCI or in cases where the patient experienced acute coronary syndrome (ACS), necessitating unplanned coronary angiography within two years (Figure 1). The patients were categorized into TLR and non-TLR groups during the follow-up angiography.

The inclusion criteria comprised patients aged ≥ 18 years, with evidence of ischemia, presenting with either stable angina pectoris or ACS and diameter stenosis of coronary vessels ≥70%, and having received successful DCB treatment [defined as patients with TIMI flow >3, with no apparent coronary dissection (i.e., type C or above) and residual stenosis ≤30% post the initial DCB procedure], whom underwent follow-up angiography of either planned or unplanned reason within 2 years. The exclusion criteria comprised patients who did not comply with dual antiplatelet therapy after the first angioplasty and those who were lost to follow-up during the evaluation of major adverse cardiovascular events (MACE) every three months until follow-up angiography. In addition, patients with an indeterminate DCB site on follow-up angiography because of the possibility of stent failure proximal to the DCB segment area (i.e., in the hybrid case) were also excluded.

All procedures involving human participants in this study were approved by the ethical committee of Dr. Hasan Sadikin General Hospital, Bandung, West Java, Indonesia (LB.02.01/X.6.5/19/2023) and were conducted in accordance with the Declaration of Helsinki. The requirement for written informed consent was waived, given the observational retrospective study design by the aforementioned ethical committee.

### Devices and intervention protocol

Choice of vascular access, guide catheter, guidewire, balloon, or whether DCB only or hybrid approach with DES combination were left to operator's discretion. The IVI use and administration of glycoprotein IIb/IIIa receptor inhibitors were also left to the operator's discretion. To achieve a stenosis of ≤30%, balloon coronary pre-dilatation was uniformly performed in all patients. The choice of device size and post-dilatation treatment was based on the operator's decision. Balloon pre-dilatation was performed using uncoated balloons to achieve a balloon-to-vessel ratio of 0.8–1.0. The intervention procedure was performed according to the international and Asia-Pacific consensus recommendations for DCB treatment (9). Intravascular imaging was conducted before predilatation, except in cases where the IVI catheter was unable to cross the lesion, predilatation using a small balloon size [2.0 mm semi-compliant balloon (SCB)] was performed before advancing the IVI catheter. In the IVI group, lesion preparation and choice of DCB size were tailored based on morphological assessment and calculation of the reference vessel size using IVI. Patients received standard dual antiplatelet therapy (DAPT) before the procedure. They continued for at least six months in cases where only DCB was used and 12 months in the hybrid approach involving both DCB and DES, especially in ACS subsets. Only paclitaxel-coated balloons (SCB SeQuent Please; B. Braun, Melsungen, Germany) were employed at our center. Intravascular imaging was conducted using optical coherence tomography (OCT; Dragonfly OPTIS, Abbott Vascular) or intravascular ultrasonography (IVUS; Opticross, Boston Scientific).

### Angiographic data, definitions, outcomes, and clinical follow-up

Lesion characteristics and complexity were defined by the standard definition outlined by the American Heart Association/American College of Cardiology (AHA/ACC) in 2020 (10). Multivessel disease (MVD) was characterized by more than one major epicardial vessel with >70% stenosis. Lesion length was determined using the length of the DCB or DES or the sum if more than one device was utilized. Vessel diameter measurements included the distal reference diameter, minimum lumen area (MLA), minimum stent area (MSA) in DES, external elastic membrane (EEM) at the target lesion using OCT or IVUS assessment in patients who underwent IVI. In cases without IVI, the optimal diameter of the DCB used was recorded (11, 12). Notably, baseline information and angiographic characteristics were obtained from angiography reports and registries.

The primary objective of this study was to assess the incidence of TLR based on The Academic Research Consortium-2, defined as clinically driven repeat percutaneous intervention of the target lesion or bypass surgery of the target vessel performed for restenosis or other complication of DCB-treated segment restenosis angiographically within the in-segment area, which encompassed the balloon-treated segment and extended 5-mm proximal and distal to the treated area, as determined by visual estimation during follow-up angiography (13). Clinically driven revascularization was determined during follow-up angiography, indicating restenosis ≥ 50% with at least one of the following: symptoms or objective signs of ischemia presumably related to the target vessel; abnormal results on invasive functional diagnostic testing (fractional flow reserve) or stenosis ≥ 70%, even in the absence of ischemic signs or symptoms. This study investigated the incidence and potential associated factors influencing its occurrence. Information regarding TLR was derived from the DCB registry and medical and angiography records. A team of certified interventional cardiologists, blinded to clinical outcomes, meticulously reviewed all angiographic data related to TLR.

All patients in the DCB registry underwent clinical follow-up following the index procedure via telephone interviews every three months and outpatient clinic visits until follow-up angiography. The secondary objectives of this study include the MACE, defined by composite of TLR and nonfatal myocardial infarction (MI) occurring from the initial procedure until the follow-up angiography. MACE was assessed on a patient basis (Table 1) while TLR were evaluated on a lesion basis (Table 2).

**Table 1:** Baseline clinical characteristics in according to the TLR and non-TLR lesion group.

	Total patient (*N* = 80)	TLR (*N* = 11)	Non-TLR (*N* = 69)	*p*-Value
Male	55 (68.8)	7 (63.6)	48 (69.6)	0.733
Age, years	61.0 (±9.2)	60.3 (±5.8)	61.1 (±9.6)	0.779
Diabetes mellitus	26 (32.3)	6 (54.5)	20 (29.0)	0.093
Hypertension	57 (71.3)	9 (81.8)	48 (69.6)	0.497
Dyslipidemia	32 (40.0)	4 (36.4)	28 (40.6)	1.000
Smoker	39 (48.8)	7 (63.6)	32 (46.4)	0.343
Family history of premature CAD	17 (21.3)	3 (27.3)	15 (20.3)	0.701
Known HF	14 (17.5)	2 (18.2)	12 (17.4)	1.000
LVEF, %	53.3 (±12.2)	52.2 (±11.3)	53.4 (±12.4)	0.756
LVEF < 40%	10 (12.5)	1 (9.1)	9 (13.0)	1.000
Previous MI	40 (50.0)	5 (45.5)	35 (50.7)	0.745
Previous PCI	47 (58.8)	7 (63.6)	40 (58.0)	1.000
Previous Stroke	2 (2.5)	0	1 (2.9)	1.000

Data was derived from patient based data (overall *N* = 80 patients). *p*-value represents association between TLR and non-TLR group and baseline characteristics of the study.

ACS, acute coronary syndrome; CAD, coronary artery disease; CCS, chronic coronary syndrome; HF, heart failure; LVEF, left ventricular ejection fraction; MI, myocardial infarction; PCI, percutaneous coronary intervention; TLR, target lesion revascularization.

**Table 2:** Procedural and lesion characteristics, lesion preparation, DCB characteristic and post DCB complication.

	All lesion (*N* = 100)	TLR (*N* = 11)	Non-TLR (*N* = 89)	*p*-Value
Indication of angiography				
Clinical presentation during initial procedure				0.420
Chronic coronary syndrome	89 (89.0)	9 (81.8)	80 (89.9)	
Acute coronary syndrome	11 (11.0)	2 (18.2)	9 (10.1)	
Reason of angiography evaluation				0.250
Acute coronary syndrome	3 (3.0)	1 (9.1)	2 (2.2)	
Staging or symptomatic chronic coronary syndrome	42 (42.0)	6 (54.5)	36 (40.4)	
Angiography evaluation	55 (55.0)	4 (36.4)	51 (57.3)	
Procedural characteristics				
Puncture site (*n*, %)				
Radial	50 (50.0)	6 (54.5)	44 (49.4)	0.671
LDTR	6 (6.0)	0	6 (6.7)	
Femoral	44 (44.0)	5 (45.5)	39 (43.8)	
Number vessel disease				0.702
1 vessel disease	5 (5.0)	0	5 (5.6)	
2 vessel disease	15 (15.0)	2 (13.3)	13 (86.7)	
3 vessel disease	80 (80.0)	9 (11.3)	71 (79.8)	
Multivessel disease (≥2 VD)	95 (95.0)	11 (100)	84 (94.4)	1.000
Reference vessel diameter, mm	2.9 (0.74)	2.8 (1.16)	3.0 (0.67)	0.143
Non-small vessel (≥2.75 mm)	77 (77.0)	8 (72.3)	69 (77.5)	0.721
Target DCB coronary lesion				
LAD	44 (44.0)	4 (36.4)	40 (44.9)	0.877
LCX	35 (35.0)	5 (45.5)	30 (33.7)	
RCA	16 (16.0)	2 (18.2)	14 (15.7)	
RI	2 (2.0)	0	2 (2.2)	
LMCA	3 (3.0)	0	2 (3.4)	
ISR	20 (20.0)	4 (36.4)	16 (18.0)	0.223
DeNovo lesion	80 (80.0)	7 (63.6)	73 (82.0)	0.223
Ostial lesion	48 (48.0)	4 (36.4)	44 (49.4)	0.529
Calcified lesion	51 (51)	3 (27.3)	48 (53.9)	0.118
CTO	13 (13.0)	1 (9.1)	12 (13.5)	1.000
Bifurcation	32 (32.0)	4 (36.4)	28 (31.5)	0.742
ACC/AHA lesion type				
A	1 (1.0)	0	1 (1.4)	0.335
B1	7 (7.0)	0	5 (7.2)	
B2	37 (37.0)	6 (60.0)	22 (31.9)	
C	55 (55.0)	4 (40.0)	41 (59.4)	
Lesion complexity				0.337
High (ACC/AHA	54 (54.0)	4 (36.4)	50 (56.2)	
lesion type C) Non-high	46 (46.0)	7 (63.6)	39 (43.8)	
Lesion preparation				
IVI utilization	**80** (**80)**	**5** (**45.5)**	**75** (**84.3)**	**0**.**002**
IVI type				
None	20 (20)	6 (54.5)	14 (15.7)	0.010
IVUS	53 (53)	3 (27.3)	50 (56.2)	
OCT	27 (27)	2 (18.2)	25 (28.1)	
Pre-dilation procedure				
Pre-dilation balloon type				0.518
Semi-compliant and non-compliant balloons	40 (40.0)	3 (27.3)	37 (41.6)	
Semi-compliant balloon	17 (17.0)	2 (18.2)	15 (16.9)	
Non -compliant balloon	23 (23.0)	1 (9.1)	22 (24.7)	
Modified balloon	60 (60.0)	8 (72.7)	52 (58.4)	
Scoring balloon	18 (18.0)	2 (11.1)	16 (18.0)	
Cutting balloon	42 (42.0)	6 (54.5)	36 (40.4)	
Pre-dilation device diameter, mm	2.5 (0.50)	2.5 (0.50)	2.5 (0.44)	0.396
Pre-dilatation device length, mm	13 (5)	15 (5.0)	14 (6.0)	0.345
Pre-dilation pressure, atm	15 (6)	18 (6)	14 (6)	0.211
Rotational atherectomy	13 (13.0)	2 (18.2)	11 (12.4)	0.633
DCB characteristic				
DCB only	32 (32.0)	5 (45.5)	27 (30.3)	0.311
Number of DCB used				
1 DCB	82 (82.0)	8 (72.7)	74 (83.1)	0.698
2 DCBs	12 (12.0)	2 (18.2)	10 (11.2)	
3 DCBs	6 (6.0)	1 (9.1)	5 (5.6)	
DCB diameter, mm	3.0 (0.5)	3.0 (1.0)	3.0 (0.5)	0.833
DCB to reference vessel ratio	0.95 (0.11)	1.00 (0.11)	0.95 (0.10)	0.349
DCB length, mm	25 (15.0)	30 (15.0)	25 (17.5)	0.379
Diffuse long lesion (>60 mm)	18 (18.2)	1 (9.1)	17 (19.3)	0.683
DCB inflation time, second	60 (0)	60 (0)	60 (0)	0.406
DCB maximal inflation pressure, atm	8 (6)	10 (4.0)	8 (7)	0.467

Data was derived from lesion based data (overall *N* = 100 lesions). *p*-value represents association between TLR and non-TLR group and baseline characteristics of the study.

CTO, chronic total occlusion; DCB, drug coated balloon; LAD, left artery descending; LDTR, left distal trans radial; LCx, left circumflex artery; LMCA, left main coronary artery; RCA, right coronary artery; RI, ramus intermedius; ISR, in-stent restenosis; IVI, intravascular imaging; TLR, target lesion revascularization.

Bold value aims to point *p* value < 0.05 (significant value).

### Statistical analysis

All statistical analyses were performed using SPSS (version 24.0; SPSS Inc., Chicago, IL, USA). Data were presented as mean ± standard deviation (SD) or median (interquartile range, IQR), and dichotomous variables were represented as counts and percentages. Baseline patient characteristics, lesion features, and procedural details (such as lesion length, reference vessel diameter, pre-dilatation balloon diameter, pre-dilatation inflation pressure, DCB diameter, and DCB length) were analyzed and compared based on the incidence of TLR. Continuous variables were compared using the independent Student's *t*-test and Mann–Whitney *U*-test, while categorical variables were assessed using Fisher's exact or chi-squared test, as appropriate. The statistical significance level for the bivariate analysis was set at 0.05. Primary and secondary outcomes were analyzed using Fisher's exact test or the chi-squared test, and the odds ratio (OR) was also calculated. Subgroup analysis was performed to explore lesion characteristics using IVI. Odds ratios (ORs) with 95% confidence intervals (CIs) were computed using a multivariate logistic regression model, incorporating variables with *p*-values < 0.25 in the univariate analysis to identify the predictors of restenosis.

## Results

### Baseline characteristics

Among the 80 patients who underwent angiographic evaluation, 100 lesions were evaluated for TLR, which was found in 11 cases (11.0% among 100 lesions) and 11 patients (13.8% among 80 patients) and all underwent revascularization of either PCI or bypass surgery. The mean age of the patients was 61.0 (±9.2) years, with 68.8% (*n* = 55) being male. The angiographic evaluation was conducted at a median of 151 (IQR: 109) days, and there was no significant difference in the number of days to follow-up angiography between the TLR and non-TLR groups (195 [IQR: 114] vs. 147 [IQR: 100]; *p* = 0.123). Common cardiovascular risk factors included hypertension (71.3%), dyslipidemia (40.0%), smoking (48.8%), diabetes mellitus type II (32.3%), a family history of premature coronary artery disease (CAD) in 21.3%, and a history of MI (50.0%). The mean left ventricular ejection fraction was 53.3% (±12.2). Baseline characteristics for the TLR and non-TLR groups were comparable, as shown in Table 1 (*p* > 0.05).

### Target lesion and procedural characteristics

The baseline target lesions and procedural characteristics are presented in Table 2. The reference vessel diameter was 2.9 (IQR 0.74) mm, with a DCB implanted in non-small vessels (≥2.75 mm) in 77% of the lesions. However, the reference vessel diameter did not significantly correlate with TLR (*p* = 0.143). ACS was the clinical presentation during the initial procedure in 11.0% (*n* = 11) of lesions and during follow-up angiography in 3.0% (*n* = 3). Among the three patients who underwent angiography during follow-up for ACS, all three presented with non-ST-segment elevation MI, and only one patient showed a post-DCB lesion as the culprit lesion.

The proportion of MVD (≥2 VD) did not differ between the TLR and non-TLR groups (*p* = 1.000); 15% of all lesions had two-vessel disease, and 80% had three-vessel disease. Neither lesion location nor the complexity of the lesion, including chronic total occlusion (CTO), ostial, calcified, and bifurcation lesions, showed an association with TLR on follow-up (*p* > 0.05). Fifty four (54%) lesions were classified as high-complexity lesions, as defined by the ACC/AHA as type C. The complexity of the lesion (type C vs. type B2 or below) was not associated with TLR events (40.0% vs. 59.4%, *p* = 0.335). DCBs were employed to treat ISR in 20% of the lesions (*n* = 20), and 20% developed TLR (*n* = 4). The TLR rate among the 80 *de novo* lesions treated with DCB was 8.7% (*n* = 7).

DCB-only treatment was administered in 32% of the lesions (*n* = 32), while the remaining involved DES implantation as hybrid approach during the index procedure. There was no observed association between the choice of DCB-only treatment or the hybrid approach involving DES and the occurrence of TLR (*p* = 0.311) (Table 2). In addition, there were no report of pericardial effusion, cardiac tamponade, or coronary perforation post-DCB implantation.

### Lesion preparation, device characteristics

**Figure 2:**
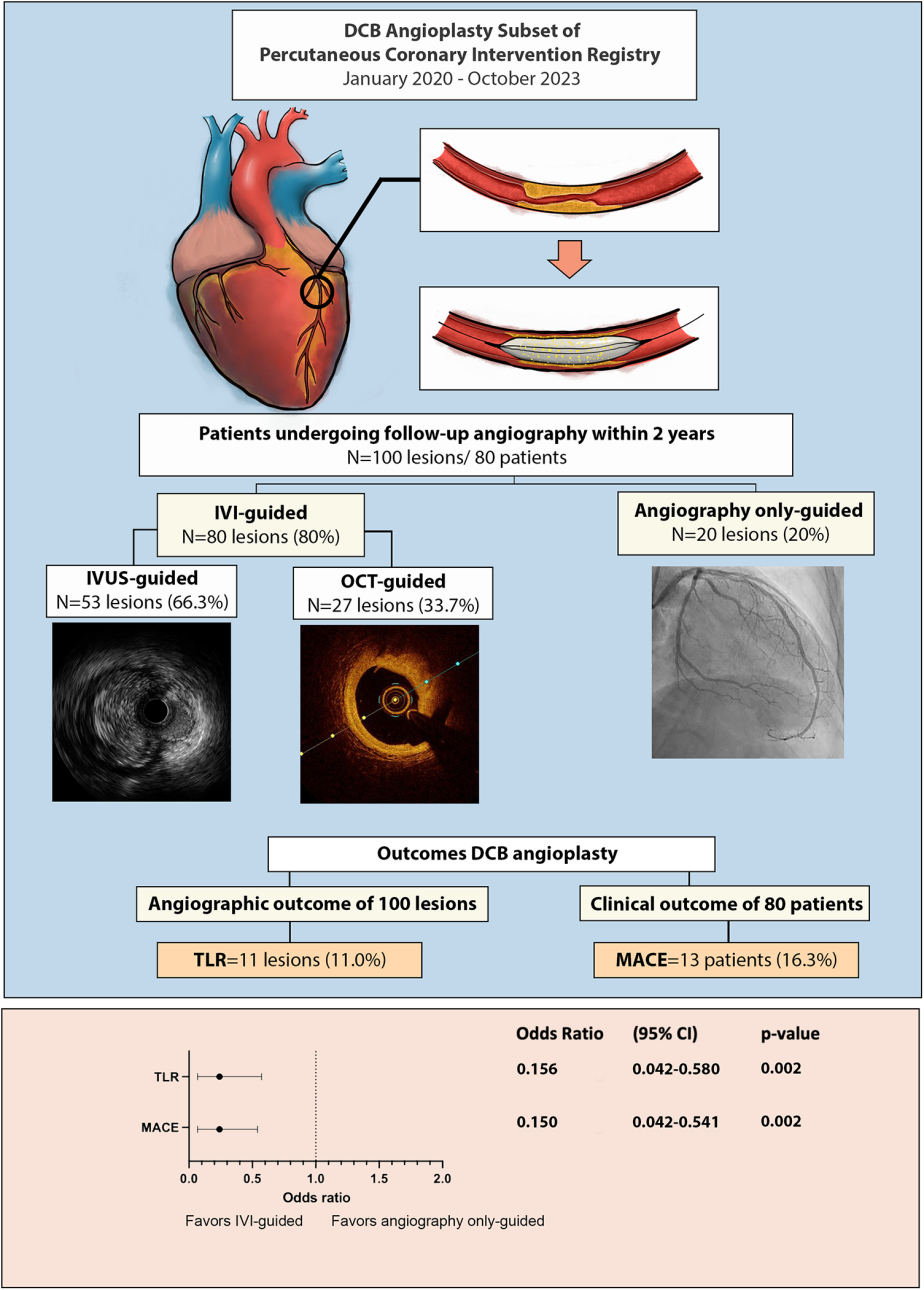
Central illustration: outcome of drug coated balloon angioplasty and the importance of intravascular imaging.

Intravascular imaging, including IVUS and OCT, was conducted for 80 lesions (80%) before DCB angioplasty. Balloon pre-dilatation was uniformly performed in all lesions, in which a modified balloon incorporating a cutting and scoring balloon was used more frequently than a semi-compliant or non-compliant balloon (60% vs. 40%). Subgroup analysis of lesion preparation balloons revealed significantly higher utilization of modified balloons in calcified vs. non-calcified lesions (70.6% vs. 49.0%; OR: 2.5, 95% CI: 1.098–5.691; *p* = 0.027). No association between the TLR and non-TLR group was found between diameter, length, maximal balloon inflation pressure, and pre-dilatation balloon type (Table 2). In the IVI group, subgroup analysis revealed significantly higher IVI use in highly complex, calcified, ostial, and non-small-vessel lesions (p < 0.05). Other lesion characteristics did not differ substantially between the IVI and non-IVI groups (Table 3). Despite the higher complexity of lesions in the IVI group, subgroup analysis demonstrated that IVI-guided PCI was associated with a lower rate of TLR on follow-up compared to angiography alone (6.3% vs. 54.5%; OR: 0.156, 95% CI: 0.042–0.580; *p* = 0.002) Figure 2. Multivariate logistic regression model analysis showed that IVI use was independently associated with a lower TLR rate (adjusted OR: 0.116, 95% CI: 0.020–0.669; *p* = 0.016), as presented in Table 4. No differences in lesion characteristics, as defined by IVI, were observed in either the *de novo* or ISR groups (Table 5) regarding TLR events.

**Table 3:** Lesion characteristic based on intravascular imaging utilization.

Baseline lesion characteristics	Imaging (*n* = 80)	No imaging (*n* = 20)	*p*
Lesion complexity			**0**.**004**
High (ACC/AHA lesion type C)	49 (61.3)	5 (25.0)	
Non-high	31 (38.8)	15 (75.0)	
Vessel diameter			**<0**.**001**
Small vessel (<2.75) mm	12 (15.0)	11 (55.0)	
Non-small vessel (≥2.75 mm)	68 (85.0)	9 (45.0)	
ISR lesion	15 (18.8)	5 (25.0)	0.532
De novo lesion	65 (81.3)	15 (75.0)	0.532
Calcified lesion	50 (62.5)	1 (5.0)	**<0**.**001**
CTO lesion	9 (11.3)	4 (20.0)	0.287
Multivessel disease	76 (95.0)	19 (19.0)	1.000
Bifurcation lesion	27 (33.8)	5 (25.0)	0.453
Ostial lesion	44 (55.0)	4 (20.0)	**0**.**006**
Diffuse lesion (≥60 cm)	17 (21.3)	1 (5.0)	0.112
Lesion preparation			**0**.**031**
Modified balloon (scoring and cutting balloon)	53 (66.3)	8 (40.0)	
Semi-compliant and non-compliant balloons	27 (33.8)	12 (60.0)	

*p*-value represents association between IVI and non-IVI group and baseline characteristics of the study.

CTO, chronic total occlusion; ISR, in-stent restenosis; IVI, intravascular imaging.

Bold value aims to point *p* value < 0.05 (significant value).

**Table 4:** Independent predictors of TLRs on multivariate analysis.

	Unadjusted OR (95% CI)	Adjusted OR	*p*-Value
IVI utilization	**0.216** (**0.062–0.758)**	**0.116** (**0.020–0.669)**	**0**.**016**
DM	2.245 (0.634–7.950)	3.099 (0.710–13.534)	0.133
ISR lesion	2.607 (0.681–9.980)	1.948 (0.394–9.626)	0.413
Calcified lesion	0.320 (0.080–1.287)	1.114 (0.177–6.988)	0.909

*p*-value represents association between TLR and non-TLR group and baseline characteristics of the study.

DM, diabetes mellitus; ISR, in-stent restenosis; IVI, intravascular imaging.

Bold value aims to point *p*-value < 0.05 (significant value).

**Table 5:** Plaque morphology in *de novo* and ISR lesion in DCB angioplasty with IVI utilization.

Plaque morphology	Total (*N* = 80)	TLR (*N* = 5)	Non-TLR (*N* = 75)	*p*
De novo lesion				0.550
Calcified	46 (57.5)	3 (60)	43 (57.4)	
Fibrotic	19 (23.8)	0	19 (25.3)	
ISR lesion				0.568
Stent underexpansion	6 (7.5)	1 (20)	5 (6.7)	
Neointimal hyperplasia	5 (6.3)	0	5 (6.7)	
Calcified neotherosclerosis	1 (1.3)	0	1 (1.3)	
Non-calcified neoatherosclerosis	3 (3.8)	1 (20)	1 (1.3)	

Data was derived from lesion treated with IVI (overall *N* = 80 lesions). *p*-value represents association between TLR and non-TLR group and plaque characterization based on intravascular imaging in *de novo* and ISR group.

DCB, drug coated balloon; ISR, in-stent restenosis; IVI, intravascular imaging.

### Intravascular imaging usage, clinical and angiography outcome

Not all restenosis cases were clinically significant; 36.4% of all restenosis lesions were found in routine angiography evaluation. MACE, defined as TLR and/or ACS, occurred in 13 (16.3%) patients. Two out of three patients with ACS did not have the culprit lesion related to the post-DCB site. The usage of IVI was also associated with lower clinical outcomes defined by MACE vs. angiography-alone group (9.5% vs. 41.2%; OR: 0.150, 95% CI: 0.042–0.541; *p* = 0.002).

## Discussion

This is a cohort study with a follow-up period of approximately two years (median, five months) of angiography after DCB PCI conducted to capture the real-world scenarios where DCB was employed in various clinical settings, particularly in cases of MVD, either as a treatment for *de novo* or ISR lesions. Additionally, we also explored the use of DCB-only or the hybrid approach. This study has contributed to the growing trend in DCB usage, highlighting the importance of accumulating more experience in real-world clinical practice. Such experience holds promise for future strategies.

### Incidence of TLR in DCB treatment

The observed rates of TLR in this study were higher than the other studies. For instance, in a study by Lee et al., which involved 2,666 coronary artery lesions treated with DCB, TLR occurred in only 5.1% of their patients (14). Our study also demonstrated higher restenosis rates than that in the PEARL registry, a real-world analysis of DCB use in the Netherlands conducted by Vlieger et al. In their study of 513 patients treated with DCB, the incidence of TLR was 11.7% in ISR lesions and 2.9% in *de novo* lesions (15). We considered that the main reason for this discrepancy was identification of most restenosis cases during follow-up angiography or staging procedures, regardless of the symptoms. Moreover, adopting a more liberal routine angiography follow-up strategy in our center might have resulted in more reintervention cases. Another factor could be the higher proportion of MVD in our study population compared to the Lee et al. registry study, where only 65.2% of patients had MVD. Shin et al. investigated the clinical impact of DCB on MVD and TLR in 3.1% of the DCB group, with follow-up angiography and subsequent revascularization being clinically driven rather than planned, regardless of symptoms. Contrary to their findings, clinically driven angiography due to ACS was observed in only 3% of the lesions in our current study (16). The current study includes all patients undergoing follow-up angiography because of both clinically driven and planned angiographic evaluation strategies for complex lesions, potentially leading to higher angiography findings, including clinically silent lesions. However, there might be unaccounted factors that could have influenced the restenosis rate.

### Complexity lesion, lesion preparation, and intravascular imaging usage association with TLR event

The hybrid DES/DCB approach dominated lesion revascularization in our study (68%), with TLR rates similar to those observed in DCB only approach lesion (*p* = 0.311). So were complexity lesion, as it also did not show difference in TLR rate between complex and non-complex lesion. The lack of a statistically significant association between lesion complexity and TLR in this study suggested that lesion complexity did not impede the utilization of this approach. Costopulous et al. supported the hybrid DES/DCB approach in lesions with a high restenosis risk, demonstrating comparable MACE and TLR at the 2-year follow-up compared to the DES-only strategy (17). This hybrid strategy holds promise in reducing the need for extensive stent implantation, especially in long lesions, thereby minimizing the risk of ISR while maintaining effective antiproliferative action and potentially reducing the need for future reinterventions. A substudy of the HYPER trial further supported the use of DCB as an adjuvant to DES, showcasing its safety and effectiveness in revascularizing coronary bifurcation lesions (18). Even in challenging cases, such as CTO lesions, DCB emerged as a feasible and well-tolerated treatment with a low rate of MACE after successful revascularization with balloon angioplasty (19). Additionally, in the study by Nagai et al. focusing on a stentless strategy for calcified lesions following rotational atherectomy, TLR rates were comparable to those in our study, with no cardiac deaths reported at an average of 196 ± 37 days after the initial PCI (20). Regardless of lesion complexity, achieving optimal angiography results after pre-dilatation (i.e., residual stenosis < 30%, TIMI grade 3, and no flow-limiting dissection) was reported to be crucial before DCB implantation (18). Therefore, emphasizing optimal lesion preparation is essential to reduce future adverse events after DCB intervention, including IVI.

In this study, IVI utilization was significantly higher in the non-TLR group. Eighty percent lesion was treated with IVI during lesion preparation and the TLR was significantly lower in the IVI group (6.3% vs 54.5%; OR: 0.156, 95% CI: 0.042–0.580; *p* = 0.002). The improvement in coronary blood flow before DCB application was achieved through adequate pre-dilatation to induce dissection and facilitate homogenous drug delivery. Aside from attaining optimal lesion preparation by either non- or semi-compliant balloon or usage of non-compliant or scoring/cutting balloon as predilatation balloon and atherectomy in more complex lesions (i.e., calcified lesions), additional IVI such as IVUS and OCT is recommended (5, 21). Moreover, satisfactory balloon angioplasty pre-DCB implantation results are usually achieved with a vessel ratio of 0.8–1.0, which is more likely to be precise using imaging. This study revealed no significant difference in plaque morphology, as assessed by IVI, between the TLR and non-TLR groups. Thus, optimizing PCI by determining the precise stent sizing and landing zones might be more critical rather than solely focusing on the underlying mechanism of plaque morphology or the type of morphology itself. Intravascular imaging can contribute to a more accurate diagnosis of minor dissection and residual stenosis (22–24). Guidance using IVI has been recommended, particularly for ISR lesions, to identify and correct the morphological causes of lesion failure (Class IIa recommendation) (25). Research has also suggested that additional IVIs should be considered for *de novo* lesions, especially in more complex cases (9). However, a propensity-matched study by Lv et al. failed to demonstrate a significant difference in target lesion failure between the IVI-guided and angiography-guided groups. Notably, their study included clinical outcomes as endpoints, such as death and MI, in contrast to our study, which specifically focused on the angiographic outcomes of DCB angioplasty, where MI occurred in only 3% (22).

In our study, IVI utilization was higher than in a previous study by Lv et al. (80.0% vs. 7.8%). Intravascular imaging was predominantly employed in 49 out of 54 high-complexity lesions or type C ACC/AHA lesions, which was significantly higher than in 31 out of 46 non-high-complexity lesions (i.e., type B2 or less) (90.7 vs. 67.3%; *p* = 0.004) (22). The number of calcified and ostial lesions was also significantly higher in the IVI group (Table 3). Intravascular imaging offers a more comprehensive and accurate calcification assessment and provides optimal guidance for lesion preparation in calcified lesions, and it may also help resolve the ambiguity of vessel overlap in ostial lesions (24). Despite the higher complexity of lesions in the IVI group, the TLR rate was lower in the IVI group than in the angiography-alone group. This suggests that using IVI to guide lesion preparation is crucial, particularly for complex lesions.

Other lesion preparation components, such as choice of balloon angioplasty and calcium modification by atherectomy, did not exhibit significant differences between the TLR and non-TLR groups. However, this should not indicate that balloon angioplasty, DCB size selection, or atherectomy planning did not impact lesion preparation before DCB implantation. A sub-study analysis indicated that modified balloon utilization was significantly higher in calcified lesions. Therefore, the lack of a significant association in this study may suggest that the appropriateness of aggressiveness in lesion preparation before DCB implantation, supported by IVI, is crucial to ensure a thorough inspection of lesion characteristics after pre-dilatation. This was consistent with a review by Basavarajaiah et al., who recommended IVI with a modified balloon as the preferred lesion preparation choice for complex calcified lesions (26). Theoretically, the local tissue drug distribution of DCB should be superior after more pronounced neointimal modification with a scoring balloon, as observed in the RCT study by Kufner et al. In that study, DCB pre-dilatation using a scoring balloon in 203 patients with ISR showed a lower rate of angiographic restenosis in the scoring balloon pre-dilatation arm compared to that via standard therapy. However, it is worth noting that the previous study used quantitative coronary angiography for lesion preparation in all subjects, while our study primarily utilized IVI, which could contribute to more optimal lesion preparation regardless of the choice of balloon angioplasty or atherectomy (24). Similar reasoning might also apply to the selection of DCB size and length, which did not significantly impact our study's angiographic outcomes of TLR events. In contrast, Xue et al. investigated factors influencing restenosis in ISR lesions after DCB angioplasty in 199 lesions at nine months of follow-up. They demonstrated that lesion characteristics, such as longer lesions, were associated with restenosis due to increased area exposure to vascular injury and aggravated inflammatory response. However, previous studies did not provide data on IVI use, potentially leading to errors in estimating the degree of stenosis. These studies mainly focused on the ISR population, representing only a small proportion of our study (18.8%) (8). Furthermore, this study's stentless strategy following atherectomy showed results similar to those in the Nagai et al. study, which demonstrated acceptable clinical and angiography outcomes (20).

### Limitations

This study had several limitations. Firstly, the small population size and the limited size of the follow-up population were the main limitations of the study. The use of DCB was initially limited to specific lesions such as ISR and small vessel disease, until recently, when its use expanded to include *de novo* and non-small vessel disease. Thus, it is only recently that enrollment has become faster. Secondly, the inclusion criteria in this study focused only on patients who underwent angiography follow-up without considering in-hospital mortality and death before angiography follow-up. Consequently, this study demonstrated better angiographic outcomes than clinical MACE outcomes after DCB. It is important to note that factors not accounted for in this study, such as drug compliance, lifestyle modifications after initial PCI, and environmental factors, could potentially influence TLR. Thus, further randomized controlled trials are needed to validate whether using IVI improves the outcomes of DCB PCI.

## Conclusion

The incidence of TLR in DCB angioplasty was eleven percent. Baseline characteristics such as diabetes mellitus, vessel size, and lesion complexity did not influence the TLR rate. In addition, the utilization of IVI significantly reduced the TLR rate. Therefore, our results suggest that DCB angioplasty can be considered as an option to DES for the treatment of either *de novo* or ISR in small or non-small coronary lesions, employing a DCB-only approach, or as an adjuvant tool to DES in complex lesions, with no difference in TLR rate as long as lesion preparation is optimized, which might be enhanced by IVI utilization.

## Data Availability

The raw data supporting the conclusions of this article will be made available by the authors, without undue reservation.
